# Geometry-based framework for beam angle selection in proton therapy for lung cancer

**DOI:** 10.1016/j.phro.2026.100958

**Published:** 2026-03-29

**Authors:** Kyriakos Fotiou, Per-Ivar Lønne, Eirik Malinen

**Affiliations:** a Department of Medical Physics, Oslo University Hospital, Oslo, Norway; b Department of Radiation Biology, Oslo University Hospital, Oslo, Norway; c Department of Physics, University of Oslo, Oslo, Norway; d Danish Centre for Particle Therapy, Aarhus University Hospital, Aarhus, Denmark

**Keywords:** Proton therapy, Beam angle selection, 4DCT, Intrafractional motion, WEPL, Lung cancer

## Abstract

**Background and purpose:**

Proton therapy is a promising modality for treating locally advanced non-small cell lung cancer (LA-NSCLC). However, respiratory-induced intra-fractional motion can compromise tumour coverage. This work aimed to develop a method to identify proton beam angles that balance tumour coverage and organ-at-risk (OAR) sparing.

**Materials and methods:**

An open-source dataset of eleven LA-NSCLC patients with four-dimensional CT (4DCT) scans was analysed. For each gantry-couch angle, the water equivalent path length variation (ΔWEPL) and percentage irradiated volume (PIV) were calculated using in-house code to serve as metrics for tumour motion sensitivity and geometric OAR exposure. A unified risk map was constructed based on ΔWEPL and PIV, with patient-specific weighting factors and constraints to enable individualised beam selection. Subsequent single-beam treatment plans were generated on the average intensity projection CT, with the dose distribution recalculated on all breathing phases to derive ΔD_95%_ (variability in target D_95%_). Pearson’s correlation tested associations between ΔWEPL and ΔD_95%_ and between PIV and OAR dose.

**Results:**

ΔWEPL correlated with target dose degradation (median r = 0.90 for ΔD_95%_), while PIV correlated with OAR doses (r = 0.88–0.98 across heart, lungs, and spinal cord). Risk maps identified beam angles minimising motion sensitivity while respecting OAR constraints. A three-beam patient example illustrated that clinically acceptable tumour coverage was maintained while substantially reducing lung and heart dose compared to suboptimal beam orientations.

**Conclusions:**

Patient-specific proton beam angle selection by combining ΔWEPL and PIV into a unified risk map offers a clinically relevant strategy to improve robustness and OAR sparing for LA-NSCLC.

## Highlights

• Four-dimensional images were used to guide proton beam angle selection.
• Beam geometries were evaluated for sensitivity to respiratory motion.
• Organ exposure was estimated from geometric beam organ intersections.
• A risk map framework balanced tumour coverage and organ sparing.
• Validation showed agreement between geometric metrics and delivered dose.

## Graphical abstract

**Figure:**
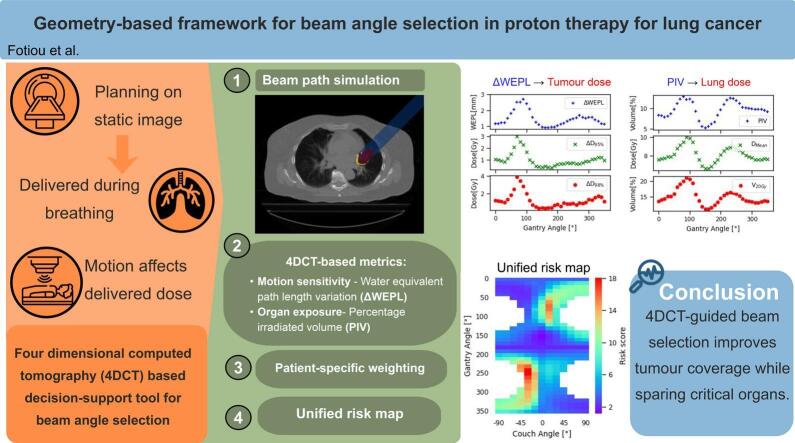

## 1 Introduction

The aggressive nature and poor prognosis of lung cancer necessitates new treatment strategies [1]. For inoperable locally advanced non-small cell lung cancer (LA-NSCLC), conventional photon radiotherapy is challenged by the tumour's proximity to organs-at-risk (OARs), making it difficult to deliver a treatment within the required dose constraints [2]. Intensity-modulated proton therapy (IMPT) presents a promising alternative, offering improved dose distribution with reduced OAR exposure. However, the efficacy of IMPT may be impeded by anatomical variations occurring both inter- and intra-fractionally [3], [4].

Intra-fractional motion during irradiation can alter the dose distribution, leading to regions of under- and over-dosage. Water equivalent path length (WEPL) analysis has been employed to evaluate such variations by comparing the planned and delivered radiological beam paths to identify optimal beam angles that minimise tumour dose degradation. WEPL variations (ΔWEPL) correlate strongly with target dose degradation, making them a key factor in beam geometry optimisation [5], [6], [7], [8], [9]. Moreover, an optimal beam selection in proton therapy must balance tumour coverage with OAR avoidance [10], [11]. Studies have shown that factors such as tumour location and patient-specific variables, such as smoking or comorbidities, influence the severity of OAR adverse events, emphasising the need for personalised treatment planning [12], [13].

This study aimed to develop a fast, stand-alone method utilizing 4DCT images to identify optimal beam angles for proton therapy of lung cancer patients, using metrics for motion sensitivity and geometric OAR exposure, independent of a treatment planning system. The method calculated target ΔWEPL and the percentage irradiated volume (PIV) of key OARs as geometry-based indicators. These quantities were used to construct personalized risk maps identifying beam geometries associated with reduced motion sensitivity and geometric OAR exposure. The method was validated against single-beam treatment plans based on the same 4DCT information.

## 2 Material and methods

### 2.1 Patient dataset

To assess the efficacy of our angle optimisation methodology, eleven LA-NSCLC patients were analysed. Patients exhibited varying tumour sizes, motion amplitudes, and locations, making the dataset clinically representative (see Supplementary Material, Table S1). The patients were selected from The Cancer Imaging Archive 4D Lung Dataset, where the gross tumour volume (GTV) and OARs had been outlined in all 10 breathing phases [14], [15]. A 5 mm isotropic expansion defined the clinical target volume (CTV), and the internal target volume (ITV) was defined as the union of all CTVs across the breathing cycle. For OARs, a composite contour encompassing all respiratory phases was used.

### 2.2 Tumour and organ-at-risk dose metrics

An in-house algorithm was developed in Python v3.9.13. To identify optimal beam geometries, two predictive metrics were considered; ΔWEPL to characterise motion sensitivity and PIV to estimate geometric OAR exposure. The patient CT images were converted to relative stopping power (RSP) maps using a Hounsfield lookup table generated through stoichiometric calibration, as described by Schneider et al. [16]. Subsequently, WEPL values were calculated by integrating the RSP values along the proton beam paths. For each unique gantry-couch angle, the algorithm simulated proton beam paths to the distal edge of the CTV. Multiple proton rays were traced per beam, and WEPL values were calculated for both the average intensity projection CT (AIP-CT) and all ten 4DCT phases. ΔWEPL was defined as the mean absolute difference in WEPL between the AIP-CT and each 4DCT phase, averaged across all rays and breathing phases for a given beam orientation. In parallel, the PIV of the investigated OARs, i.e., heart, lungs, and spinal cord, was quantified. PIV represents the fraction of an OAR volume intersected by the ray-traced beam path and serves as a geometry-based surrogate for organ exposure. ΔWEPL and PIV were calculated across gantry angles from 0°–360° (10° intervals) and couch angles from -90°–90° (15° intervals). Non-deliverable angle combinations were excluded. A total of 350 unique gantry-couch angle combinations were investigated per patient. The algorithm is available in the accompanying GitHub repository: (https://github.com/FotiouK/Geometry-based_framework_for_beam_angle_selection_in_proton_therapy_for_lung_cancer).

### 2.3 Beam angle selection

The selection of favourable incident proton beam angles requires a balance between tumour coverage and OAR exposure. To systematically evaluate the delivery space, the calculated metrics (ΔWEPL or PIV) were mapped for each volume of interest (VOI) as a function of gantry and couch angles to create two-dimensional risk maps. To effectively integrate both ΔWEPL and PIV into a single metric, we employed Z-score normalization of each risk map (see Supplementary Material, Section A). Following Z-score normalisation, the risk maps represent relative risk values reflecting the sensitivity of beam geometries across VOIs, rather than absolute clinical dose constraints. Each VOI risk map could additionally be assigned an individual weighting factor, analogous to weighting strategies used in treatment planning optimisation, allowing the treatment planner to prioritise structures. In addition to weighting, hard constraints on selected OARs could be imposed by excluding beams that intersect the structure or exceed a user-defined PIV threshold. The normalised risk maps, once weighted and adjusted for constraints, were then summed to create a final unified risk map. This map thus combined the WEPL- and PIV-based risk information (with planner-defined adjustments), enabling identification of beam angles with lower relative risk. The optimal set of a user-defined number of beams can then be selected based on the lowest risk scores. To reduce overlap between incident beams, a minimum beam separation criterion was applied using the central angle theorem (see Supplementary Material, equation S3).

### 2.4 Validation plans

Proton therapy plans for validation were created in RayStation 12A (RaySearch Laboratories, Stockholm, Sweden) with the 4DCT image series employed above. The AIP-CT with ITV and composite OARs across the breathing cycle was employed as the planning CT. Thirty-six single-beam spot scanning plans for each patient were optimised non-robustly, covering every 10° gantry angle in the interval from 0° to 360°, where couch angle was set to 0°. All plans were normalised to a median ITV dose of 66 Gy. All patients were planned with a conventional 33-fraction scheme using institution-specific dose-volume objectives for LA-NSCLC, consistent with clinical practice at our centre.

The 4D dose distribution was estimated by recalculating the treatment plan on all 10 breathing phases of the 4DCT scan. The dose distribution from each phase was deformably transferred back to the planning CT and averaged, assuming an equal contribution from each breathing phase. Tumour dose degradation was recorded through inspection of the ITV D_95%_ and D_98%_ reduction from the optimised plan and was defined as,

(1) $$\Delta D_{x\%}=D_{x\%}^{\mathrm{Planned}}-D_{x\%}^{4D}$$

where $D_{x\%}$ is the dose deposited to x% of the volume in Gy. Additionally, OAR dose-volume statistics were extracted. Finally, the association between the ΔWEPL metric with tumour dose degradation and PIV with OAR accumulated dose was analysed and quantified utilising Pearson’s correlation coefficient (r).

### 2.5 Patient example

To illustrate the functionality of the proposed in-house methodology, three scenarios were developed for a three-beam IMPT patient case, each with different beam-selection objectives. For scenario 1, weighting factors were applied to minimise tumour dose degradation (via ΔWEPL) and dose to the heart and lungs (via PIV), with less emphasis on the spinal cord. The specific weights used to construct the final risk map were 2 for the tumour, 1.5 for the heart and lungs, and 0.5 for the spinal cord. Scenario 2 employed the same weighting factors but introduced a constraint to exclude beams intersecting the spinal cord. Scenario 3 represented a benchmark for suboptimal planning, utilising the least-optimal beam configuration identified in scenario 1, corresponding to the beam geometries that maximised the risk score. Scenario 3 was used to demonstrate that high risk scores correlate with poor treatment plan quality. For each scenario, the final risk map was constructed, and a set of three beams was identified, with a minimum separation of 20° between each beam.

Subsequently, 4D robustly optimised treatment plans for the patient in question were generated in RayStation with a 5 mm isotropic setup uncertainty and a ± 3.5% range uncertainty, incorporating all respiratory phases. Dose evaluation employed the 4D robust individual phase evaluation (4DRobInd) method [17]. This approach generated 21 dose distributions per phase, totalling 210 across all respiratory phases. To account for intra-fractional motion, the average value from the dose volume histogram (DVH) across all breathing phases for each parameter combination was used. Tumour coverage was assessed using the worst-case scenario D_98%_ deviation from the planned (D_98%, robust_), while OAR doses were evaluated through maximum heart D_Mean_, lungs D_Mean_, and spinal cord D_5%_.

## 3 Results

**Fig. 1:**
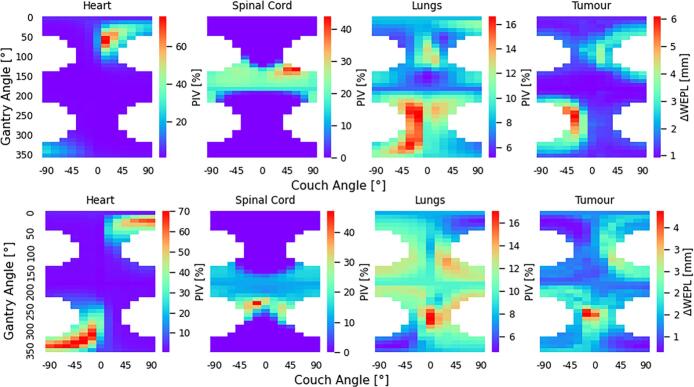
Risk maps for OAR percentage irradiated volume (PIV) and tumour mean ΔWEPL maps for patient A (top) and patient B (bottom) for all deliverable beam configurations. For both OAR PIV and tumour ΔWEPL, smaller values reflect a more favourable beam angle.

Risk maps quantified and visualised favourable beam geometries by combining motion sensitivity (ΔWEPL) and geometric OAR exposure (PIV) (Fig. 1). Distinct variations between patients were observed, reflecting differences in tumour location, proximity to OARs, and respiratory motion.

**Fig. 2:**
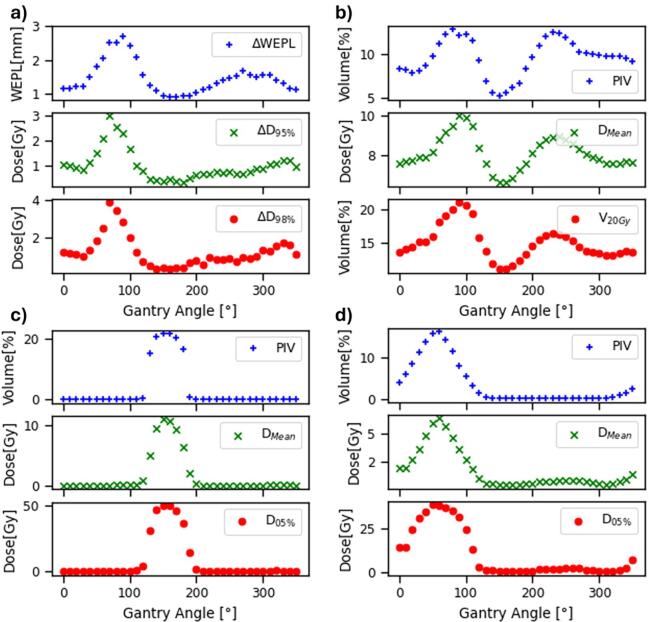
Dependence of gantry angle on ΔWEPL and PIV from the proposed in-house algorithm and dose/volume metrics from the validation plans in RayStation for Patient A. (a) Target (ITV), (b) lungs, (c) spinal cord, and (d) heart.

For Patient A, ΔWEPL and PIV showed strong correlations with target and organ dose metrics across gantry angles (Fig. 2). Pearson’s correlation coefficients were 0.87 and 0.85 between ΔWEPL and ΔD_95%_ and ΔD_98%_, respectively. Correlations between PIV and organ dose metrics were 0.98 and 0.97 for heart D_5%_ and D_Mean_, 0.99 and 0.98 for spinal cord D_5%_ and D_Mean_, and 0.90 and 0.87 for lung D_Mean_ and V_20 Gy_ (percentage volume receiving > 20 Gy), respectively.

**Fig. 3:**
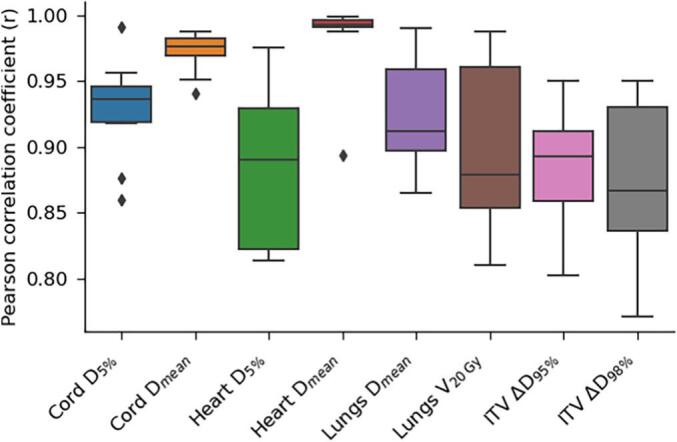
Cohort-based correlations between metrics from the in-house algorithm (ΔWEPL for target, PIV for OAR) and ITV dose degradation and OAR dose parameters from the validation plans. Box plots depict the median, and first and third quartiles.

Across the entire patient cohort, the metrics from our in-house algorithm maintained strong correlations with tumour and OAR dose metrics from the validation plans (Fig. 3). ΔWEPL exhibited a strong positive correlation with ΔD_95%_ and ΔD_98%_, with a population median r of 0.90 and 0.87 and interquartile ranges (IQR) of 0.05 and 0.09, respectively. Similarly, PIV showed a strong positive correlation with OAR dose, with median correlations for OAR dose statistics ranging from 0.88 to 0.98 and IQRs from 0.01 to 0.11.

**Fig. 4:**
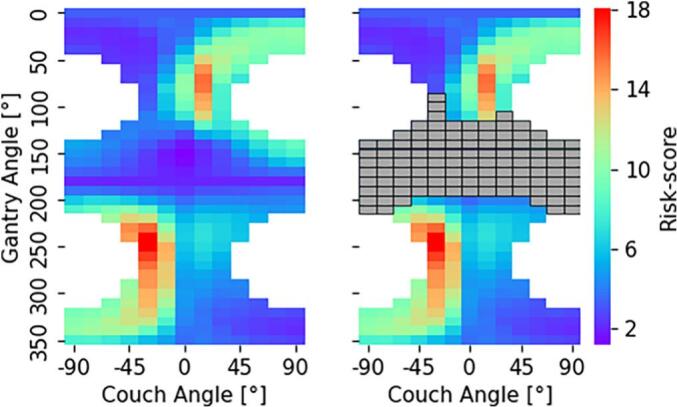
Unified risk maps for patient A for scenarios 1 and 3 (left) and scenario 2 (right). Restricted geometries due to spinal cord irradiation are depicted in grey*.*

For Patient A, the selected gantry-couch angle configurations were (150°, 0°), (190°, 0°), and (20°, -60°) for Scenario 1; (30°, -45°), (20°, -90°), and (50°, -45°) for Scenario 2; and (70°, 15°), (240°, -30°), and (260°, -30°) for Scenario 3, based on the unified risk maps depicted in Fig. 4.

**Fig. 5:**
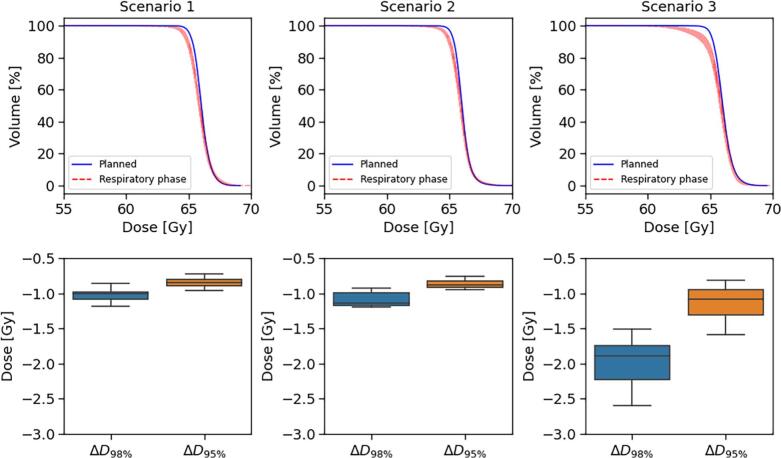
Target DVHs from robust plan optimisation for three-beam IMPT patient case, shown for each treatment scenario with proton beam configurations selected from our in-house method (top). The planned dose distribution is portrayed in blue, with the worst-case respiratory phases depicted in red. Below, box plots depict the corresponding distribution of the ΔD_95%_ and ΔD_98%_ across the ten respiratory phases. (For interpretation of the references to colour in this figure legend, the reader is referred to the web version of this article.)

For the three-beam IMPT patient case, robust treatment planning with the in-house predicted beam configurations from scenarios 1 and 2 yielded similar tumour coverage performance (Fig. 5), with worst-case ΔD_98%_ values of 1.0 Gy and 1.1 Gy, respectively. Conversely, scenario 3 demonstrated substantially degraded target coverage, with a ΔD_98%_ of 2.4 Gy, which indicated higher susceptibility to intrafractional motion. Additionally, the observed IQR for ΔD_98%_ across breathing phases, were 0.2, 0.3, and 0.7 Gy for scenarios 1, 2, and 3, respectively. Nominal dose distributions for each treatment plan are provided in Supplementary Material, Fig. S1.

**Fig. 6:**
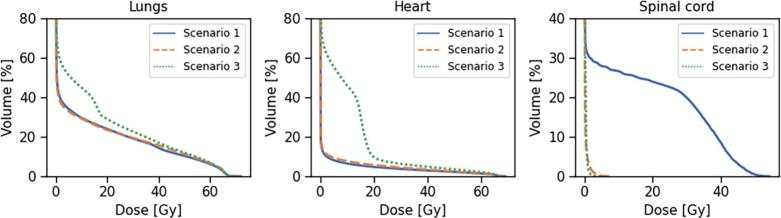
DVHs for lungs (left), heart (middle), and spinal cord (right) from robust treatment planning for the three-beam IMPT patient case, shown for the three scenarios with proton beam configurations selected from our in-house method.

Evaluation of DVHs for OARs showed marked reductions in lung and heart doses for scenarios 1 and 2 compared to scenario 3 (Fig. 6). For instance, the lung D_Mean_ was reduced from 50 Gy in scenario 3 to 36 Gy and 37 Gy in scenarios 1 and 2, respectively. Similarly, the heart D_Mean_ was reduced from 32 Gy to 8 Gy and 10 Gy, respectively. Spinal cord D_05%_ was 42 Gy in scenario 1, but was reduced to 0.5 Gy for scenario 2 due to the hard constraints on beams intersecting the spinal cord in this case. Furthermore, the spinal cord dose in scenario 3 was only 0.5 Gy. This reflected the applied OAR weighting scheme in scenario 1, where heart and lung PIV were assigned weights of 1.5 and the spinal cord a weight of 0.5. As scenario 3 corresponded to beam geometries with the highest risk score under this weighting, configurations less favourable for the heart and lungs resulted in comparatively lower spinal cord dose. Still, across all scenarios, the imposed weights on ΔWEPL and PIV effectively predicted the dosimetric impact of the selected beam geometries on the corresponding volumes.

## 4 Discussion

This study presented a geometry-based approach for identifying favourable proton beam geometries for lung cancer by considering target coverage and OAR exposure. In single-beam spot scanning treatment plans, ΔWEPL from our in-house method showed strong correlations with tumour dose degradation metrics, while PIV of the heart, lungs, and spinal cord served as a surrogate metric reflecting geometric OAR exposure. The three-beam IMPT patient case illustrated that beam angles selected by the proposed method yielded favourable tumour coverage and reduced OAR doses under robust optimisation. This example was intended to demonstrate methodological functionality rather than to provide a validation of the algorithm’s metrics in multi-field IMPT. Our framework was designed as a geometry-based beam angle pre-selection step to reduce solution space prior to robust optimisation. Within this scope, the novelty of the approach lies in its flexibility to empirically balance competing planning objectives during beam selection in a patient-specific manner.

Analysis of ΔWEPL maps across the patient cohort revealed variations influenced by tumour size, location, and breathing-induced anatomical changes. Favourable beam orientations were often posterior, oblique-posterior, and oblique-anterior. These beams typically avoided extensive tissue paths and minimised the intersection with high-WEPL dynamic structures, both of which increase susceptibility to range variations. In contrast, cross-lateral and right-left configurations often showed higher WEPL due to these anatomical factors. These findings are consistent with prior studies that identified posterior beams as optimal for tumour dose coverage [5], [6], [8], [9].

Expanding upon the work of Casares-Magaz et al. [7], which focused exclusively on ΔWEPL without accounting for OARs, our study assessed a full range of gantry-couch angles in a larger patient cohort. Our analysis revealed significant limitations of using ΔWEPL alone for beam angle selection. Specifically, utilizing the ΔWEPL map alone to select three beams, with a minimum 20° separation, we observed that 9 out of the 11 patients had at least one beam intersecting the spinal cord. This demonstrated that minimising motion sensitivity often comes at the expense of OAR sparing. To address this, our methodology integrated additional OAR metrics to guide angle selection. While OAR sparing has been incorporated in beam angle selection for head and neck tumours using an alternative approach [18], our study is the first to demonstrate a methodology that simultaneously balances competing target and OAR objectives during the angle selection process. Unlike fully automated or deep-learning-based approaches [19], [20] our approach incorporated user-defined priorities, allowing the planner to exert direct control over the trade-off between motion resilience and OAR sparing. Given that tumour location and patient-specific factors influence OAR adverse events [12], [13], this flexibility may enable adaptation of beam angles to the individual’s clinical need. While our study focused on the lungs, heart, and spinal cord, the approach could be extended to incorporate additional critical organs such as the trachea and esophagus.

The present study investigated proton therapy of patients in free breathing without the incorporation of motion management techniques such as beam gating, deep inspiration breath-hold (DIBH), or abdominal compression [21], [22], [23]. Their application is evaluated on an individual basis, depending on tumour location and the patient’s capacity for stable breathing or sustained breath-holds. Motion management techniques can be integrated into the proposed methodology by utilising, e.g., gated phases or DIBH motion-extended CT scans. This allows identification of optimal beam geometries while accounting for refined motion provided by these techniques.

By design, the proposed in-house framework omitted a physical model of the proton dose deposition, excluding effects such as lateral scattering and beam divergence. The derived ΔWEPL and PIV metrics were intended to quantify the relative influence of beam geometry on tumour motion sensitivity and organ exposure to effectively narrow the vast gantry-couch search space before dose optimisation. This simplified approach enabled high computational efficiency, allowing rapid evaluation of all deliverable gantry-couch angle combinations without the need for dose calculation. The methodology was intended to be applied upstream of treatment planning to identify a limited set of patient-specific, geometrically favourable beam geometries that can subsequently be used as input for robust multi-field optimisation in a commercial treatment planning system. As clinical delivery constraints and imaging workflows vary between institutions, practical limitations such as couch mobility, collision avoidance, and patient immobilization (commonly with both arms above the head) [6], [21], [24] may limit the feasibility of certain orientations, including anterior-oblique beam configurations. Such factors can be incorporated as user-defined restrictions to maintain clinical applicability of the framework.

A limitation of our study is the exclusion of interplay effects, which result from the dynamic interaction between the tumour motion and delivery of proton beam spots. These effects can potentially lead to under- or over-dosing of the target [25]. Our investigation focused solely on radiological path differences due to breathing-induced variations, which resulted in Bragg peak shifts and subsequent target dose degradation. The established methods to minimize interplay effects, such as rescanning and repainting, are independent of beam angle [26]. Consequently, these techniques do not influence the geometric selection of beam angles, which remains the primary focus of this study. Additionally, interplay effects have been reported to be more prominent in hypo-fractionated treatments and are reduced in fractionated regimens [27], [28] such as the 33-fraction treatment employed in our study. Therefore, evaluating interplay effects was deemed beyond the scope of this work.

In conclusion, this study implemented a new beam selection methodology based on surrogate metrics of motion sensitivity and geometric OAR exposure in proton therapy for lung cancer. By balancing competing geometric objectives, our approach identified beam geometries with reduced sensitivity to intra-fractional motion. These findings were benchmarked against single-field spot scanning plans. With further validation and the inclusion of additional thoracic OARs, this methodology may serve as a decision-support tool for beam arrangement in proton therapy.

## 5 Declaration of generative AI and AI-assisted technologies in the manuscript preparation process

During the preparation of this manuscript, the authors used ChatGPT (GPT-5.3, OpenAI) to assist with improving the clarity and readability of the English text. The tool was used only for language refinement. All scientific content, analyses, and interpretations were conceived, verified, and validated by the authors. The manuscript was carefully reviewed and edited after the use of this tool, and the authors accept full responsibility for the final published content.

## CRediT authorship contribution statement

**Kyriakos Fotiou:** Writing – review & editing, Writing – original draft, Visualization, Validation, Software, Methodology, Investigation, Formal analysis, Data curation, Conceptualization. **Per-Ivar Lønne:** Writing – review & editing, Validation, Supervision, Methodology. **Eirik Malinen:** Writing – review & editing, Validation, Supervision, Methodology, Conceptualization.

## Declaration of competing interest

The authors declare that they have no known competing financial interests or personal relationships that could have appeared to influence the work reported in this paper.
