# Supplementary material for: Geometry-based framework for beam angle selection in proton therapy for lung cancer
Source: Phys Imaging Radiat Oncol. 2026 Mar 29;38:100958. doi: 10.1016/j.phro.2026.100958 (PMC13089173; doi:10.1016/j.phro.2026.100958)
Supplement: Supplementary data 1 [file mmc1.pdf]

Supplementary Material

Section A: Dataset

This section includes additional information regarding the dataset used in this study. Tumour motion and volume were estimated using an in-house Python v3.9.13 algorithm, the code for which is available in the GitHub repository (https://github.com/FotiouK/Tumor-Motion-Analysis). Tumour motion was assessed through a vector deformation algorithm, while the average tumour volume was calculated based on the delineated gross tumour volumes (GTVs) from all respiratory phases of the 4DCT. Table S1 portrays the geometric and motion information for all patients employed in our study.

Table S1: Patient characteristics and tumour motion parameters. (RUL =right upper lung, RLL = right lower lung, LUL = left upper lung, LLL = left lower lung)

Patient	Tumour location	Tumour motion [mm]	Average tumour volume [cm^3]
A	RUL	2.3	70
B	LLL	4.0	51
C	RUL	10.9	28
D	LUL	9.9	183
E	LLL	5.6	37
F	Mediastinum	13.3	150
G	LUL	3.3	19
H	RUL	11.1	11
I	RLL	10.4	57
J	RLL	9.4	79
K	RLL	5.1	187

Section B: Risk maps and Z-score normalisation.

To integrate the information from the water equivalent path length variation (ΔWEPL) and organ-at-risk (OAR) percentage irradiated volume (PIV) maps, Z-score normalisation was employed. This statistical approach standardised the pixel values of all maps, facilitating their integration into a unified metric. The Z-score equation is expressed as,

$$Z = \frac{x_i - \mu}{\sigma} \qquad \text{(S1)}$$

where x_i is the value of the ith pixel element of the risk map corresponding to a unique gantry-couch angle combination, μ is the mean and σ the standard deviation of the risk map population. Z-score transformation was applied to every pixel of the risk maps, as illustrated in Figure 1 in the main text, converting pixel values into relative variables indicative of the deviation from the population average in terms of standard deviation.

To establish a comprehensive singular metric, all risk maps were multiplied by varying weighting factors and aggregated to generate a unified risk (UR) map. The mathematical formulation for a given pixel of the UR map is defined as,

$$x_i^{UR} = \sum_Z W_Z x_i^Z = W_T x_i^T + W_H x_i^H + W_L x_i^L + W_S x_i^S \qquad \text{(S2)}$$

where $\mathrm{W_Z}$ denotes the weighting factor assigned to the plan parameter Z. Plan parameters assessed in our study include the tumour (T), heart (H), lungs (L) and spinal cord (S).

For the selection of optimal incident beam geometries, the central angle theorem was employed to impose restrictions to avoid cross beam irradiation. The central angle theorem describes the angular separation of two points located on the surface of a sphere, which in our case represent two arbitrary gantry positions while the sphere centre represents the treatment isocentre. Mathematical formulisation of the central angle (CA) theorem is defined as,

$$\mathrm{CA} = \arccos\left(\sin(\mathrm{GA_1}) \sin(GA_2) + \cos(\mathrm{GA_1}) \cos(GA_2) \cos(|CA_1 - CA_2|)\right) \qquad \text{(S3)}$$

where subscripts 1 and 2 represent the gantry (GA) and couch angle (CA) of the two assessed beam geometries.

Section C: Dose distributions for the three scenario plans

This section depicts the nominal dose distributions for the treatment plans generated according to the methodology described in Section 2.5 of the main text. The corresponding dosimetric analysis is reported in the Results, Section 3. Each treatment plan consisted of three beam angles identified using our proposed beam-angle selection framework, with scenario-specific objective weighting factors and constraints.

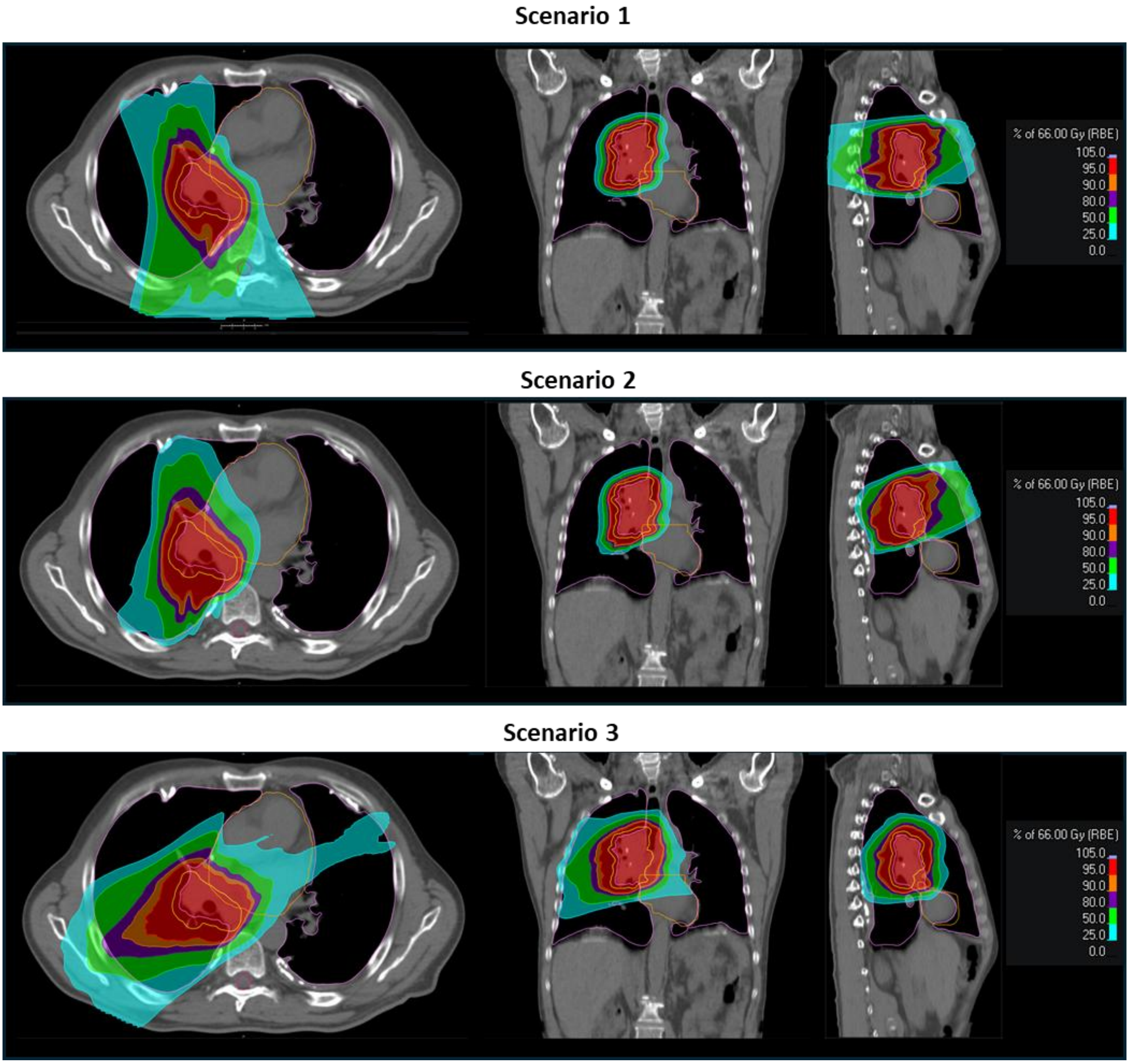

Figure S1: Axial (left), coronal (middle), and sagittal (right) views of the nominal dose distributions for the three beam-selection scenarios for the representative patient described in Section 2.5. The colour wash represents dose as percentage of the prescribed dose of 66 Gy (RBE).
